# Procedure providing SI-traceable results for the calibration of protein standards by sulfur determination and its application on tau

**DOI:** 10.1007/s00216-022-03974-z

**Published:** 2022-03-22

**Authors:** Nora Lemke, Ahmed H. El-Khatib, Teodor Tchipilov, Norbert Jakubowski, Michael G. Weller, Jochen Vogl

**Affiliations:** 1 Bundesanstalt für Materialforschung und -prüfung (BAM), Richard-Willstätter-Straße 11, 12489 Berlin, Germany; 2 Charité - Universitätsmedizin Berlin, Hessische Str. 3-4, 10115 Berlin, Germany; 3 Department of Pharmaceutical Analytical Chemistry, Faculty of Pharmacy, Ain Shams University, Cairo, Egypt; 4 Spetec GmbH, Am Kletthamer Feld 15, 85435 Erding, Germany

**Keywords:** Inductively coupled plasma mass spectrometry, Isotope dilution, Quantitative protein analysis, Sulfur, SI traceability

## Abstract

Quantitative proteomics is a growing research area and one of the most important tools in the life sciences. Well-characterized and quantified protein standards are needed to achieve accurate and reliable results. However, only a limited number of sufficiently characterized protein standards are currently available. To fill this gap, a method for traceable protein quantification using sulfur isotope dilution inductively coupled plasma mass spectrometry (ICP-MS) was developed in this study. Gel filtration and membrane filtration were tested for the separation of non-protein-bound sulfur in the protein solution. Membrane filtration demonstrated a better performance due to the lower workload and the very low sulfur blanks of 11 ng, making it well suited for high-purity proteins such as NIST SRM 927, a bovine serum albumin (BSA). The method development was accomplished with NIST SRM 927e and a commercial avidin. The quantified mass fraction of NIST SRM 927e agreed very well with the certified value and showed similar uncertainties (3.6%) as established methods while requiring less sample preparation and no species-specific standards. Finally, the developed procedure was applied to the tau protein, which is a biomarker for a group of neurodegenerative diseases denoted “tauopathies” including, e.g., Alzheimer’s disease and frontotemporal dementia. For the absolute quantification of tau in the brain of transgenic mice overexpressing human tau, a well-defined calibration standard was needed. Therefore, a pure tau solution was quantified, yielding a protein mass fraction of (0.328 ± 0.036) g/kg, which was confirmed by amino acid analysis.

**Graphical abstract:**

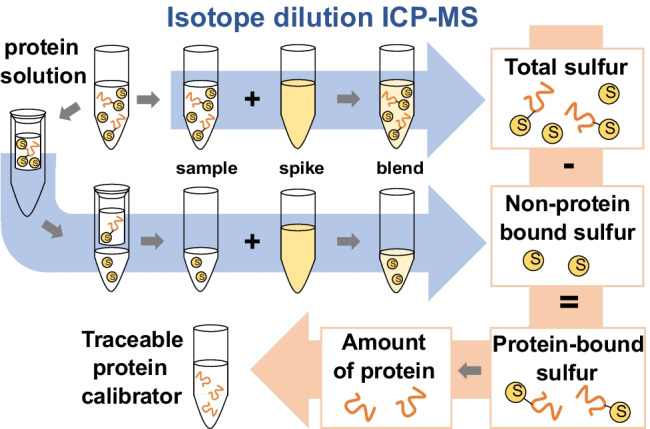

**Supplementary Information:**

The online version contains supplementary material available at 10.1007/s00216-022-03974-z.

## Introduction

The quantitative analysis of proteins has nowadays become one of the most important tools in the life sciences and has found its way into the analytical sciences together with the biological and medical questions centered around proteins [1]. Quantitative proteomics data might help to further the understanding of regulatory processes, the efficacy of drugs, the effect of biomarker interactions, and a multitude of other biological mechanisms [2]. Clinical diagnostics especially require reliable and comparable data because health-related decisions are based upon this data [3, 4]. Ensuring comparability of clinical data is crucial due to the globalized production and distribution of diagnostic devices and pharmaceuticals [5]. Comparability can only be achieved by traceability to the same source, preferably the International System of Units (SI). Therefore, well-characterized and SI-traceable protein standards are urgently needed to achieve inter-lab comparability of diagnostic procedures and research. Still, there is a pronounced lack of sufficiently characterized and quantified protein standards, and only very few metrologically traceable certified reference proteins are available [2]. Commercially available pure proteins are usually not sufficiently well characterized to enable comparability between standards. Moreover, the gravimetric determination of the protein concentration in a solution prepared from lyophilized protein usually is not possible because information on remaining water, salt content, and other impurities in the lyophilized protein is not provided by the manufacturer and can easily sum up to a significant fraction of the material.

In principle, the protein concentration in the solution can be determined by a variety of analytical methods, although most of them are not suitable due to different reasons. Conventional protein quantification methods rely on the existence of well-characterized peptide or protein standards or labeling of the protein [6–8]. For most proteins, however, no well-characterized protein standards are available, thus facing a catch-22 situation. An alternative approach without the need for protein or peptide standards is amino acid analysis (AAA). In AAA, the protein is hydrolyzed into its amino acids, which are subsequently quantified by molecular mass spectrometry using an amino acid calibrator [9]. Although AAA is considered the gold standard in accurate and traceable protein quantification, the optimization of hydrolysis conditions is critical and can strongly influence the accuracy of the result [10–12]. In the last 20 years, elemental mass spectrometry, i.e., inductively coupled plasma mass spectrometry (ICP-MS), emerged as a method for absolute protein quantification [13]. The “hard” ionization in ICP-MS is robust and species-independent [14] and enables absolute quantification of proteins via heteroatoms or labeling of the protein, e.g., with lanthanides [15]. Some excellent reviews give an overview of the existing methods [8, 16, 17]. One such method is isotope dilution ICP-MS (ID-ICP-MS). ID-ICP-MS has the potential to be applied as a high-quality primary method that can be used for traceable protein quantification, e.g., via the sulfur content of the protein. The heteroatom sulfur is present in over 98% of all proteins via the sulfur-containing amino acids cysteine and methionine [18, 19].

A very sophisticated method for traceable protein quantification based on sulfur ID-ICP-MS was developed by Lee et al. [20]. They used ID-ICP-MS for protein quantification via its sulfur content and size-exclusion chromatography coupled to ICP-MS for the separation and quantification of sulfur-containing contaminations. The method is highly accurate but also rather time-consuming and elaborate and most suited for the quantification of protein reference materials. Another sophisticated method for traceable protein quantification using species-specific ID-ICP-MS was recently published by Schaier et al. [21]. They used sulfur-containing amino acids as standards and traceably quantified amyloid β peptides by HPLC-ICP-MS, which is highly accurate but also requires hydrolysis of the sample and isotopically labeled yeast extract, which is not commercially available.

Here, we present a simplified method for the quantification of in-house protein calibrators, which can easily be applied in every ICP-MS laboratory. The ID-ICP-MS–based approach enables the SI-traceable quantification of pure proteins of known stoichiometry and includes a procedure to correct for sulfur-containing impurities. Non-protein-bound sulfur compounds were separated by an easy-to-handle and cost-effective offline filtration procedure and were quantified by ID-ICP-MS. The herein used approach is species-unspecific ID-ICP-MS, in which the spike (inorganic sulfate) has not the same molecular composition as the sample (protein). As no spike specific to a certain protein needs to be produced, the method is easily applicable to every protein of known sulfur composition. The protein quantification method was developed and tested on the certified reference material SRM 927e bovine serum albumin (BSA) from the National Institute of Standards and Technology (NIST), as well as commercially available avidin. The approach was finally applied for the quantification of an in-house calibrant for the Alzheimer’s disease biomarker tau protein, and the result was verified by aromatic amino acid analysis (AAAA), a variant of amino acid analysis, which requires no derivatization step [12].

## Materials and methods

### Reagents and materials

The certified reference material NIST SRM 3154 (0.1% H_2_SO_4_) was purchased from the National Institute of Standards and Technology (NIST, Gaithersburg, MD, USA) and was used as a sulfur backspike. Elemental sulfur enriched in ^34^S (99.8%) was obtained from Trace Sciences International Inc. (Delaware, USA) in solid form and was used to prepare the spike solution as described in Phukphatthanachai et al. [22]. Isotopic composition and atomic weight were previously reported by Pritzkow et al. [23]. l-Methionine (BioUltra grade, ≥ 99.5%) and 3-(cyclohexylamino)-1-propane sulfonic acid (CAPS, ≥ 99%) were obtained from Sigma-Aldrich (Steinheim, Germany). For method development, the bovine serum albumin solution (BSA) SRM 927e was obtained from NIST [24]. BSA is not the most suitable protein for the herein-developed method (due to high binding affinities to other compounds, possible restructuring of disulfide bonds, the reaction of free cysteine with glutathione in blood plasma). However, no other protein reference materials were currently available. To minimize the risk of restructuring, NIST SRM 927e was kept at 4 °C, and a new ampule was opened for each experiment. Avidin from egg white (BioUltra grade, ≥ 98%, Sigma-Aldrich, Steinheim, Germany) and a recombinant human tau protein (tau-441, > 90%, Anaspec, Fremont, CA, USA) were obtained as lyophilized powder. For SRM 927e BSA, a molar mass of (66,431.1 ± 0.9) g/mol and a sulfur mass fraction of 1.882% sulfur referring to BSA (39 × S atoms per molecule BSA) were reported in the certificate (highest abundant molar mass). The molar masses of 67,072 g/mol for avidin with a sulfur mass fraction of 0.956% (20 × S atoms per molecules avidin) and 45,850 g/mol for tau with a sulfur mass fraction of 0.559% (8 × S atoms per molecule tau) were taken from www.uniprot.org, and expanded uncertainties of 5% (*k* = 2) were estimated for the molar masses. The absence of other protein contaminations in the tau protein was verified by sodium dodecyl sulfate–polyacrylamide gel electrophoresis and silver staining.

Ultrapure deionized water (18 MΩ·cm) purified by a Milli-Q water purification system (Millipore gradient, Merck Millipore, Darmstadt, Germany) was used for all dilutions and cleaning procedures. Twice sub-boiled nitric acid (HNO_3_) was used as a digestion agent, for matrix separation and ICP-MS measurements. Plastic laboratory equipment was cleaned by immersing in 10% (v/v) HNO_3_ for 60 h, while multi-use PFA equipment was cleaned by boiling in at least 10% HNO_3_ for 12 h or using concentrated HNO_3_ vapors for 2 h (steam stripper traceCLEAN, MLS, Leutkirch, Germany), after which it was soaked in Milli-Q water for at least 12 h and dried in cleanroom cabinets.

### Sample preparation for ID-ICP-MS

To minimize sulfur contaminations from the laboratory environment, sample preparation was conducted in an ISO 6 cleanroom (PicoTrace), in which environmental conditions, e.g., temperature, pressure, and relative humidity, are continuously monitored and adjusted. Weighing was performed on an analytical balance (Mettler Toledo AX205, Giessen, Germany) after flushing PFA labware with a nitrogen ion stream for the removal of electrostatic charges. Sample and spike solutions were weighed by the subtraction method. Spike and stock solutions were kept under evaporation control, and dilutions could adjust for at least 2 h. Protein samples were diluted to desired target concentrations between 0.3 and 1 mg/kg sulfur by weighing and were spiked close to a 1:1 ratio with a ^34^S-enriched sulfur spike. Samples and sample-spike blends were digested after the addition of 0.5–2 mL concentrated HNO_3_ (65%) with microwave small-vessel digestion (SP-Discover with autosampler, CEM, Kamp-Lintford, Germany) using quartz tubes with PFA inliners or using a high-pressure asher (HPA-S, Anton Paar GmbH, Graz, Austria) with 15-mL quartz vessels. Samples diluted in water or HNO_3_ were digested using the microwave (6 min ramping to 200 °C, holding 6–20 min at 200 °C). Samples in a high salt matrix, demanding subsequent matrix removal, were digested with the HPA under pressurized conditions (ramping to 300 °C, holding 90 min at 300 °C). Samples were transferred to PFA beakers, dried with an open lid for 12–15 h at 150 °C on the hotplate, and dissolved in 0.028 mol/L HNO_3_ for subsequent matrix removal or in 2% (v/v) HNO_3_ for direct measuring. Non-protein sulfur contaminations were separated from the protein fraction, slightly overspiked with 40–200 µg/kg of ^34^S, and measured directly. Digestion and ICP-MS measurements were carried out in ISO 7 cleanrooms.

**Fig. 1:**
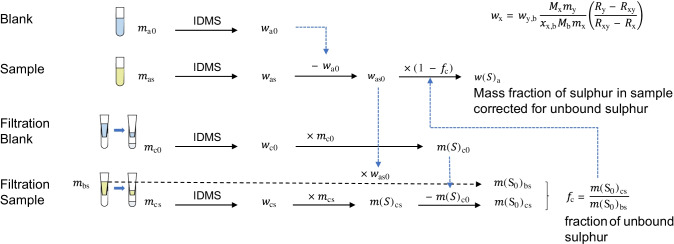
Calculation scheme for the correction of the sulfur mass fraction of a protein sample for non-protein-bound sulfur after separation by membrane filtration

Protein quantification was achieved by the determination of the total sulfur mass fraction in the sample by ID-ICP-MS, followed by separation and quantification of non-protein-bound sulfur species. The protein concentration was determined after correction for non-protein-bound sulfur (see Fig. 1). Here, it has to be noted that the protein must be free from any other protein impurities, which needs to be verified by the user and/or the producer, e.g., by applying molecular mass spectrometry or SDS-page. The associated measurement uncertainty needs to be considered. In our case, we attributed this measurement uncertainty to the theoretically calculated value of the protein mass fraction, as the SDS-page result stemmed from the producer, and we wanted to separate our measurement data and the producer’s data in the measured and the theoretical value.

### Separation of non-protein-bound sulfur

Small molecular weight sulfur species were separated from the protein fraction by gel or membrane filtration. All sample preparation steps were conducted in the cleanroom or in a clean cabinet to limit contaminations.

PD-10 gel filtration columns (GE Healthcare, Buckinghamshire, UK) containing Sephadex™ G-25 resin with a cut-off of about 5 kDa were used for the separation of protein and contaminant peaks. Elution volumes of different proteins were tested and were adapted accordingly. Sodium chloride (NaCl Suprapur, Merck KGaA, Darmstadt, Germany), 25 mmol/L, was used for column equilibration, as mobile phase, and for elution. Columns were rinsed and conditioned in 5 × 5 mL water and 5 × 5 mL 25 mol/L NaCl. Sample solution, 200–220 mg, diluted in ultrapure water was added drop by drop to the frit on top of the resin, soaked in by the addition of 300 mg water, and eluted by stepwise addition of 25 mmol/L NaCl. Protein fraction (3.2 mL for BSA, 2.6 mL for avidin) and a low molecular mass fraction (3.8 mL for BSA, 4.4 mL for avidin) were collected in cleaned falcon tubes. Samples were weighed into digestion vessels, blended with the ^34^S-enriched spike, followed by HPA digestion and matrix removal, which was carried out using self-packed anion exchange columns (AG 1-X8 resin, BioRad, Hercules, CA, USA) as described in Phukphatthanachai et al. [22]. Briefly, columns were washed with 4 × 2.5 mL ultrapure water, 2 × 2 mL 1 mol/L HNO_3_, 2 × 2.5 mL 0.25 mol/L HNO_3_, and 2 × 1.5 mL 0.028 mol/L HNO_3_. Samples dissolved in 0.028 mol/L HNO_3_ were added to the columns, allowed to bind for 30 min, washed with 2 × 2.5 mL ultrapure water, and eluted using 4 × 2 mL 0.25 mol/L HNO_3_. The eluate was dried and taken up in 2% (v/v) HNO_3_ for subsequent ICP-MS measurements.

Separation by membrane filtration was done using Amicon Ultra-0.5 centrifugal cellulose membrane filters (Merck KGaA, Darmstadt) with a cut-off of about 3 kDa. The filtration unit comprises a 1.5-mL collection tube and a 0.5-mL inner tube with a regenerated cellulose membrane. Collection tubes were rinsed with ultrapure water, immersed overnight in 5% (v/v) HNO_3_, rinsed, and soaked in ultrapure water overnight. Filters were rinsed by centrifugation for 10 min at 14,000 × *g* and 18 °C with 3 × 2% (v/v) HNO_3_, 4 × water, and 3 × 5 mmol/L ammonium hydrogen carbonate (NH_4_HCO_3_), and membranes were always kept wet. Ultrapure NH_4_HCO_3_ was produced from pure ammonia and carbon dioxide as described in Vogl et al. [25]. Samples were weighed onto the filter membrane (80–500 mg) and centrifuged for 15 min at 14,000 × *g* and 18 °C. Filtrates were transferred to PFA beakers, and filters were rinsed with 200 to 400 mg NH_4_HCO_3_. Filters with rinsing solution were shaken for 15 min at 140 min^−1^ and centrifuged for 15 min at 14,000 × *g* and 18 °C. The rinsing solution was added to PFA beakers, and rinsing was repeated 3 × . The collected filtrate was spiked with ^34^S, mixed overnight, and measured the next day directly.

### Mass spectrometry

Measurements were performed using the sector field single-collector ICP-MS Element 2 (Thermo-Fisher Scientific, Germany). Before each measurement, the instrument was tuned and mass calibrated, and the remaining mass offset for sulfur was adjusted. The used setup is shown in the ESI (Table S1). The sample introduction system consisted of an ASX520 autosampler (Cetac, Omaha, NE, USA) coupled to a 100-µL MicroMist nebulizer and a cyclonic spray chamber (both GlassExpansion, Port Melbourne, Australia). Sample aspiration was assisted by a peristaltic pump, increasing the sample consumption to 192 µL/min. Samples of natural isotopic composition were measured first, followed by sample-spike mixtures and the spike solution. High-abundant samples were measured five times, while very limited samples like mixtures of membrane-filtrated solution and spike were only measured once. In the case of purified tau protein, only 400 µg of protein was available in total. Therefore, only the isotope ratio of the sample-spike mixture was measured (3 spiking replicates), and the natural isotope ratio of the sample was determined from published data and not by ICP-MS.

### Isotope dilution ICP-MS

All calculations were carried out in Excel (Microsoft Office 365 ProPlus, Version 1902) or GUM Workbench Pro (Version 2.4.1.375, Metrodata GmbH, Weil am Rhein, Germany). As measurement sequences were up to 14 h long, instrument drift was corrected by the bracketing method using repeated measurements of a sulfur standard throughout the whole sequence.

In ID-ICP-MS, the isotope ratios ^32^S/^34^S of an analyte (*R*_x_), an isotopically enriched spike (*R*_y_), and a sample-spike blend (*R*_xy_) are used to calculate the mass fraction of the target element in the sample (*w*_x_) by applying Eq. (1). Details on the different calibration strategies and the equation system are given in Vogl et al. [26]. Here, the single ID-ICP-MS calibration approach was used.

1 $${w}_{\mathrm{x}}={w}_{\mathrm{y},\mathrm{b}}\frac{{M}_{\mathrm{x}}{m}_{\mathrm{y}}}{{x}_{\mathrm{x},\mathrm{b}}{M}_{\mathrm{b}}{m}_{\mathrm{x}}}\left(\frac{{R}_{\mathrm{y}}-{R}_{\mathrm{xy}}}{{R}_{\mathrm{xy}}-{R}_{\mathrm{x}}}\right)$$

The mass fraction of ^34^S in the spike after dilution to the target concentration (*w*_y,b_) was calculated from the known mass fraction of the spike stock solution, which has been previously determined by reverse IDMS [22, 23]. The atomic weight of the spike isotope (*M*_b_) was taken from published data [23]. The masses of the sample (*m*_x_) and spike (*m*_y_) in the blend were measured by weighing. *R*_xy_ was measured by ICP-MS, and *R*_x_ and *R*_y_ were either measured or taken from tabulated data. When only *R*_xy_ was measured, the isotope ratio was corrected for mass discrimination. The correction factor *K* was determined by measuring the sulfur standard NIST SRM 3154 and comparing the measured ratio (*R*_observed_) to the “true” isotope ratio of the standard, as shown in Eq. (2). The “true” isotope ratio is represented by the reference isotope ratio for NIST SRM 3154 with *R*_true_ = 22.555, which was determined previously by TIMS [23].

2 $$K= \frac{{R}_{\mathrm{true}}}{{R}_{\mathrm{observed}}}$$

The atomic weight of sulfur in the sample (*M*_x_) and the isotope amount fraction of ^34^S in the sample (*x*_x,b_) were taken from data published by the Commission on Isotopic Abundances and Atomic Weights (CIAAW) [27, 28] (see Table 1). For the determination of the sulfur content in protein samples, the values for organic sulfur originating from animals were used. For samples with unknown sulfur sources, e.g., contaminant fractions, the most likely sulfur sources were selected from the published data, and the most extreme interval boundaries were chosen to define an “unknown” interval. The mean of the interval boundaries was used for the ID-ICP-MS calculation, and the uncertainty was given by the halfwidth of the interval. If *R*_x_ was not measured, it was calculated from the published isotopic abundance variation expressed as the delta value (Eq. (3)) using the isotope reference material VCDT (Vienna Canon Diablo Troilite, *R*(^32^S/^34^S) = 22.6436, *δ*^34^S = 0‰ [29]) as a reference.

**Table 1:** Properties of sulfur from protein or unknown sources determined from data published by the CIAAW [27, 28]. Uncertainties *u* are given as rectangular distributions

	Molar mass of S (*M*_x_*)*/g/mol		Isotope amount fraction of ^34^S (*x*_x,b_) /mol/mol		Isotope amount ratio *n*(^32^S)/*n*(^34^S) /mol/mol	
	Mean	*u*	Mean	*u*	Mean	*u*
Protein	32.064368	0.001308	0.042192	0.000621	22.5251	0.3472
Unknown	32.063732	0.002703	0.041890	0.001282	22.7123	0.7283

3 $${\delta }^{34}S= \left(\frac{{\left(\frac{{}^{34}S}{{}^{32}S}\right)}_{\mathrm{sample}}}{{\left(\frac{{}^{34}S}{{}^{32}S}\right)}_{\mathrm{reference}}}-1\right)$$

The values needed for ID-ICP-MS calculations, which were determined from the CIAAW data, are shown in Table 1.

### Uncertainty calculation

Complete uncertainty budgets were calculated using GUM Workbench and Excel. The calculations in GUM Workbench are based on international guidelines on the evaluation of uncertainty in measurement [30]. Full uncertainty budgets for each calculated quantity, the associated uncertainties, and the contribution of all parameters are given by GUM Workbench. By using the uncertainties *u*_i_ of each sample and the standard deviation *s* of samples in *n* replicate measurements, combined standard uncertainties *u*_c_ for the mean values were calculated in Excel as shown in Eq. (4).

4 $${u}_{\mathrm{c}}=\sqrt{{\left(\frac{s}{\sqrt{n}}\right)}^{2}+\frac{\sum {u}_{\mathrm{i}}^{2}}{n}}$$

The expanded uncertainty *U* = *k · u*_c_ was calculated from *u*_c_ using a coverage factor of *k* = 2 (95% confidence).

The quantities and their uncertainties that were used for the ID-ICP-MS calculation with GUM Workbench are shown in the ESI (Table S2). Normal distribution was assumed for measured data, while rectangular uncertainty functions were used for published data for which only intervals were given.

### Correction for non-protein-bound sulfur

The determined sulfur content in the protein fraction was corrected for non-protein-bound sulfur contaminations, as shown in Fig. 1. First, the sulfur mass fraction of the sample (*w*_as_) is determined by ID-ICP-MS and is corrected for the procedure blank (*w*_a0_) to yield *w*_as0_. In the filtration procedure, the sample is weighed onto the filter (*m*_bs_) and filtered, and the filtrate is also weighed (*m*_cs_). The mass fraction of sulfur in the filtrate is determined by ID-ICP-MS (${w}_{\mathrm{cs}}$), and the total amount of sulfur (*m*(S)_cs_) is calculated from the known mass (*m*_cs_). The total mass of sulfur in the procedure blank of the filtration (*m*(S)_c0_) is determined accordingly and used for blank correction of the mass of sulfur in the filtrate (*m*(S_0_)_cs_). The total mass of sulfur in the sample before filtration (*m*(S_0_)_bs_) is calculated from the sample mass before filtration (*m*_bs_) and the sulfur mass fraction in the sample (*w*_as0_). Finally, the fraction of non-protein-bound sulfur (*f*_c_) is determined by dividing the mass of sulfur in the filtrate (*m*(S_0_)_cs_) by the sulfur mass before filtration (*m*(S_0_)_bs_). *f*_c_ is used to correct the sulfur mass fraction in the sample (*w*_as0_) to yield the protein-bound sulfur (*w*(S)_a_) from which the protein concentration in the sample is calculated.

### Aromatic amino acid analysis

Acidic hydrolysis of proteins was done using concentrated hydrobromic acid (HBr, 48% (w/w), Honeywell, Charlotte, NC, USA). Cysteine (≥ 98%, Sigma-Aldrich, Saint Louis, MO, USA), 1 mg, was dissolved in 60 µL HBr, mixed with 10 µL of the sample, sealed, and heated for 1 h at 150 °C in a hydrolysis vessel. After cooling, the hydrolyzed samples were diluted 1:5 in ultrapure water and centrifuged for 20 min at 31,000 × *g*, and the supernatant was transferred into HPLC vials.

Samples were hydrolyzed in triplicates and were injected into the HPLC instrument (Azura, Knauer, Berlin, Germany) in full loop mode (50 µL sample loop, 15 µL flush volume) using an autosampler (AS3950, Knauer, Berlin, Germany). The samples underwent chromatographic separation on a reversed-phase column (AdvanceBio Peptide Mapping, 2.1 × 150 mm, Agilent, Santa Clara, CA, USA) equipped with a guard column (AdvanceBio Peptide Mapping Guards, 2.1 × 5 mm, Agilent, Santa Clara, CA, USA). Amino acids were separated using a gradient of 10 to 90% (v/v) acetonitrile (ACN, LC–MS-grade, ≥ 99.95%, Labsolute, Renningen, Germany) in 0.2% (v/v) trifluoroacetic acid (TFA, HPLC-grade, ≥ 99.5%, Alfa Aesar, Kandel, Germany) within 4.5 min at a flow rate of 0.45 mL/min. Fluorescence of the aromatic amino acid tyrosine (Tyr) was detected at 272 nm excitation/303 nm emission (fluorescence detector RF-20Axs, Shimadzu, Duisburg, Germany). External calibration was performed using a commercial amino acid standard (analytical standard AAS18 from Supelco, Sigma-Aldrich, Saint Louis, MO, USA) containing 2.5 mmol/L l-tyrosine in 0.1 N HCl.

Fluorescence signals were integrated using Origin (OriginLab, Version 9.60), and calibration data was linearly fitted. Tyrosine concentrations of samples with their associated uncertainties were determined in Excel and GUM Workbench. The protein concentration was calculated from the known stoichiometry, and the uncertainties were determined from the uncertainties of the calibration, the standard deviation of the mean of replicate measurements, and the estimated uncertainty of the molar mass of the protein.

## Results and discussion

### Sulfur measurements

Blank values for sulfur of 4–6 × 10^4^ cps, corresponding to 6–8 ng/g, and limits of detection (LOD) of 0.2–1 ng/g (3 × blank standard deviation (SD)) were determined in Milli-Q water. Sample intensities were corrected with blanks measured directly before each sample, and drift correction was done by bracketing with sulfur standards.

Recovery tests using NIST SRM 927e BSA showed that best recoveries of the sulfur mass fraction by ID-ICP-MS were obtained after dilution of the protein by gentle shaking for 1–3 h, followed by spiking of the protein solution and digesting the samples directly afterward. Digestion has the advantage that no sample is lost due to adsorption. Moreover, sample and spike are completely blended and converted to the same molecular species (sulfate) such that both will behave equally in subsequent preparation steps and within the ICP-MS plasma. However, the amount of concentrated nitric acid (HNO_3_) used for digestion needs to be minimized because the acid contributes to the blank, and sub-boiling of the acid does not decrease its sulfur blank [23]. A sulfur blank of (25.5 ± 0.4) ng/g was found for concentrated HNO_3_ in six replicate blank digests (HPA, quartz vessels). Hence, for analyzing small amounts of samples and measuring close to the LOD, sample preparation without digestion is favorable. Here, short times between sample preparation and measuring are critical to avoid sample loss due to adsorption, which might be substantial at low concentrations. In such dilute samples, the interference of the surrounding matrix and its effect on the plasma response are almost negligible, and it is acceptable to use a spike (e.g., inorganic sulfur) of different molecular structure compared to the sample (e.g., protein) [31].

The samples were quantified with recoveries of approximately 100% even at sample-spike blend dilutions of only 20 ng/g, showing that the ID-ICP-MS approach is well suited for the quantification of low amounts of proteins. However, weighing of very low sample masses increases the relative uncertainty of the sample mass considerably and increases the impact of sample losses during preparative steps, e.g., by protein powder sticking to labware. Hence, using at least 200 ng/g of sulfur for reliable quantification of the protein concentration is suggested as a compromise between the accuracy of the result and the conservation of the precious sample.

Here, proteins with sulfur mass fractions of 0.6–1.9% were quantified. Thus, 10–35 µg of protein are needed for 1 mL of a sample solution containing 200 ng/g of sulfur. Consequently, 30–100 µg of protein are needed for three replicate measurements for protein quantification. Approximately the same amount of protein is needed additionally for the quantification of sulfur contaminations, increasing the total amount of protein needed for the analysis to 200 µg. If a very low amount of non-protein-bound sulfur is expected, the amount of protein needed to correct for contaminations can increase two- or threefold.

### Determination of non-protein-bound sulfur

Sulfur-containing salts, buffers, and organic compounds, as well as column packing material, are commonly used in protein production and purification. In commercially available proteins and even in reference materials, usually, no data for sulfur content or sulfur contaminations are given. Therefore, it is crucial for protein quantification via elemental MS to correct for non-protein-bound sulfur species. Gel filtration and membrane filtration were tested for the offline separation of low molecular weight non-protein-bound sulfur compounds. In order to test the separation and recovery of non-protein-bound sulfur from the protein fraction, 1.5 µg/g sulfate, methionine (Met), or CAPS were added to a 20-µg/g NIST SRM 927e BSA solution to yield a solution containing 0.7–1.4% of non-protein-bound sulfur.

Gel filtration has the advantage that protein and contaminant peaks can be collected, quantified, and compared within one experiment/analysis. Optimization of the gel filtration procedure showed that low amounts of sample (200–250 µL) and 25 mmol/L sodium chloride (NaCl) in the mobile phase are needed to achieve baseline separation of protein and contaminant fraction. Subsequent digestion and matrix separation were needed to remove the accumulated salt load from both fractions for the following ICP-MS measurements. These additional handling steps substantially increased the blank levels, resulting in procedure blanks of (136 ± 50) ng and (142 ± 40) ng and LODs of 393 ng and 347 ng for the protein and contaminant fraction, respectively. The amounts of unbound sulfur in pure BSA as well as BSA with added sulfate, Met, or CAPS were below the LOD and could not be determined. In pure commercial avidin, the amount of non-protein-bound sulfur contamination is much higher, as 29% of non-protein-bound sulfur was quantified after separation by gel filtration.

Membrane filtration proved to be more favorable for the separation of very low amounts of non-protein-bound sulfur. However, here, only the sulfur in the filtrate can be determined because the protein cannot be recovered from the filter quantitatively. Commercially available membrane filters containing either a polyethersulfone (PES) or cellulose membrane were tested. PES membranes strongly leach sulfur even after thorough rinsing resulting in sulfur blanks of several hundred nanograms. Cellulose filters proved suitable for the separation of sulfur impurities because only a few nanograms of sulfur were found in the blank. Recoveries of non-protein-bound sulfur were tested using sulfate, Met, or CAPS added to BSA. Met and sulfate were not completely transferred to the filtrate when water was used as the mobile phase, even after rinsing with 4 × sample volume. Better transfer of (charged) sulfur compounds to the filtrate was achieved using low amounts of salt in the mobile phase, presumably because electrostatic interactions with the membrane are shielded [32, 33], and counterions from the salt solution maintain the Donnan equilibrium across the membrane [34, 35]. An ultrapure NH_4_HCO_3_ that was produced from pure reagents was used because it decomposes to ammonia and carbon dioxide at temperatures above 60 °C [25] and therefore can be directly introduced into the plasma. A 5 mmol/L dilution was chosen as a compromise between the minimum ionic strength needed for the separation and the salt load which could be introduced into the plasma. Membrane filters with rinsing solution were gently shaken before the next centrifugation step to detach sedimented protein to minimize blocking of pores. Recoveries for non-protein-bound sulfur compounds determined by ID-ICP-MS are shown in Table 2. Good recoveries were obtained for pure sulfate and CAPS using 5 mmol/L of NH_4_HCO_3_. Recoveries determined for sulfate and CAPS added to BSA had high uncertainties caused by one outlier each. As only 80 ng of non-protein-bound sulfur was present in these samples, the outliers might have been due to slight contaminations in the sample. The differences in recoveries between pure compounds and compounds added to BSA (excluding the outlier) are presumably due to the binding behavior of BSA. BSA is a serum protein with high non-specific binding affinities because it serves as a transporter for a multitude of different molecules [36–38]. Hence, some sulfate and CAPS might have been captured by the BSA. Next, the effect of salt on the separation of non-protein-bound sulfur species was tested using commercial avidin. The recoveries obtained with membrane filtration with 5 mmol/L NH_4_HCO_3_ agreed very well with the gel filtration result, demonstrating the applicability of the procedure and the need for additional electrolytes in the mobile phase.

**Table 2:** Recoveries of sulfur-containing compounds in the filtrate after membrane filtration using different mobile phases. Uncertainties: standard deviation of replicate measurements (*N* = 3). BSA: NIST SRM 927e, Met: methionine

	Recovery of non-protein-bound S in filtrate/%	
Sample	Milli-Q water	5 mmol/L NH_4_HCO_3_
SO_4_^2−^	51 ± 9	96 ± 3
CAPS	n.a	97 ± 1
BSA + SO_4_^2−^	26 ± 26	118 ± 51 (88 ± 1)^b^
BSA + Met	73 ± 10	n.a
BSA + CAPS	99 ± 15	156 ± 132 (80 ± 7)^b^
Avidin^a^	1 ± 1	103 ± 0

^a^Recovery relative to gel filtration result.

^b^Brackets: result excluding one outlier.

*n.a.* not analyzed

The optimized filtration procedure comprised thorough rinsing of the filters, filtering 200–500 µL of sample solution, followed by 4 × rinsing with gentle shaking in between, spiking of the pooled filtrate, and direct measuring. Digestion was not carried out because no subsequent matrix removal was required, and the additional handling steps and digestion procedure might have introduced higher blanks. The procedure blanks contained (11.1 ± 1.3) ng sulfur, and the LOD and limit of quantification (LOQ) were 19.5 and 39.0 ng sulfur, respectively. The fractions of non-protein-bound sulfur in BSA and avidin are shown in Table 3. In the BSA solution, only 0.4% of the total sulfur was non-protein-bound sulfur, which shows the high purity of SRM 927e and the suitability for calibration and validation purposes. Avidin, however, contained 30% of non-protein-bound sulfur, showing that separation and correction for sulfur contaminations are crucial for the reliable quantification of commercial proteins.

**Table 3:** Fractions of non-protein-bound sulfur determined in BSA (NIST SRM 927e) and avidin after separation by membrane filtration. Uncertainty (*U*): combined uncertainty of the standard deviation and the uncertainties of each measurement (*N* = 3), given as expanded uncertainty (*k* = 2)

	Mass of non-protein-bound S ± *U*/ng	Mass of total S in sample ± *U*/ng	Fraction of non-protein-bound S in sample ± *U*/%
BSA	37.0 ± 27.9	10,047 ± 261	0.37 ± 0.28
Avidin	112.0 ± 8.3	375 ± 15	29.8 ± 2.5

### Protein quantification by sulfur ID-ICP-MS

For protein quantification, NIST SRM 927e BSA solution was diluted, and the solid avidin formulation was dissolved in 5 mmol/L NH_4_HCO_3_. After the amount of sulfur in the dilute protein solutions was quantified and corrected for non-protein-bound sulfur (0.37% for BSA, 29.8% for avidin), the sulfur content of the BSA stock solution and the crystalline avidin formulation were calculated and are shown in Table 4. The relative standard deviations of the BSA (*N* = 7) and avidin (*N* = 3) measurements were 2.2% and 3.5%, respectively. Sample preparation, e.g., handling variations during weighing or blending, had the highest impact on the variability between samples. When only small amounts of sample are available, as was here the case for avidin, weighing is challenging as the powder sticks to plastic labware due to electrostatic forces. The expanded uncertainties of the sulfur mass fractions, which include the variability of sample preparation, also reflect this problem. The final relative expanded uncertainty of BSA amounts to 3.1% for the sulfur mass fraction, whereas the quantified value for the sulfur mass fraction of avidin has a relative expanded uncertainty of 4.7%.

**Table 4:** Sulfur and protein mass fraction in BSA (NIST SRM 927e) stock solution and lyophilized avidin powder. Results with expanded uncertainties *U* with *k* = 2. BSA: 4 separate sample preparations, avidin: 3 separate sample preparations

	Replicate	*w*_x_(S)_Stock_ ± *U* /g/kg		*w*_x_(Protein)_Stock_ ± *U* /g/kg		*w*_x_(Protein)_theor_ ± *U*/g/kg
		Single value	Mean	Single value	Mean	
**BSA**	1–1	1.253 ± 0.033	1.245 ± 0.038	66.5 ± 1.8	66.1 ± 2.0	66.2 ± 1.4
	1–2	1.218 ± 0.032		64.7 ± 1.7		
	1–3	1.249 ± 0.033		66.3 ± 1.8		
	2–1	1.277 ± 0.033		67.8 ± 1.7		
	2–2	1.279 ± 0.033		67.9 ± 1.8		
	3	1.218 ± 0.031		64.7 ± 1.7		
	4	1.219 ± 0.031		64.8 ± 1.7		
**Avidin**	1	6.99 ± 0.40	6.76 ± 0.32	731 ± 56	707 ± 65	866 ± 86
	2	6.52 ± 0.44		682 ± 57		
	3	6.76 ± 0.48		707 ± 62		

By using the known molar masses of the proteins and the known number of sulfur atoms, the protein mass fractions were calculated from the sulfur mass fraction. Table 4 shows the determined sulfur and protein mass fractions compared to the theoretical protein mass fractions. The mass fraction of BSA in the stock solution was determined to be (66.1 ± 2.0) g/kg. Avidin in the lyophilized powder was determined to be (707 ± 65) g/kg, corresponding to a protein content of 70.7% in the solid avidin formulation.

The theoretical protein mass fraction of BSA and its uncertainty were calculated from the density and concentration of the stock solution given in the NIST certificate. The uncertainty of the theoretical value is slightly lower than the uncertainty of one replicate ID-ICP-MS determination and one-third lower than the uncertainty of the mean. To ascertain whether the theoretical and the measured values are metrologically compatible, the *E*_n_ value (formerly denoted as normalized error) was calculated as described in Vogl et al. [39] (see ESI 1.1). The *E*_n_ value of the theoretical and measured BSA mass fraction was determined to be 0.02, implying that the difference between the values is only 2% of the associated uncertainty and that the BSA mass fractions agree within their uncertainties.

The theoretical protein mass fraction of avidin (86.6%, 866 g/kg) was calculated from the purity ≥ 98% (0.99 ± 0.01) and protein fraction of 80–95% (0.875 ± 0.075) given by the manufacturer. The avidin mass fraction determined by ID-ICP-MS is lower than the theoretical value. However, the theoretical mass fraction is only a rough estimate because the actual protein content in the solid powder can vary greatly. The residual water content of a lyophilized protein is usually 1–5% [40, 41], and stabilizers and additives like buffers, counterions, other salts, protease inhibitors, chelating, and reducing agents [42, 43] can comprise a considerable amount of the mass. Therefore, solid protein formulations cannot directly be used as calibrators as weighing will result in incorrect results. Usually, the amount of salts and moisture is not provided by the manufacturer, and the theoretical protein content is often overestimated, as it was the case for avidin. Hence, quantifying the protein concentration in solution is necessary.

### Uncertainties and traceability

**Fig. 2:**
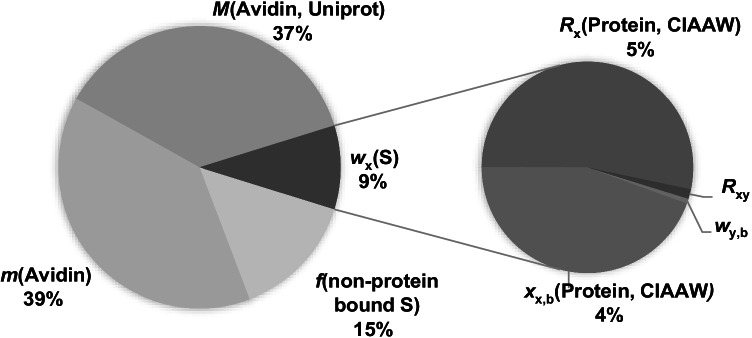
Contributors to the uncertainty of the avidin mass fraction. *R*_x_(Protein, CIAAW) and *x*_x,b_(Protein, CIAAW) were taken from tabulated data published by CIAAW. The quantities are given as intervals, and uncertainties were determined as rectangular functions of these intervals. The molar mass of avidin *M*(Avidin, Uniprot) was taken from the Uniprot database, and a high uncertainty of 5% was estimated for this quantity

The relative uncertainties of the NIST SRM 927e and avidin protein mass fractions were 3.1% and 9.2%, respectively (see ESI, Table S3). The uncertainty of every single measurement was for both proteins higher than the standard deviation of all measurements. The uncertainty of a single measurement of the NIST SRM was considerably lower (2.6%) than that of avidin (8.3%). The individual uncertainty contributors for NIST SRM 927e are shown in the ESI (Fig. S1) and for avidin in Fig. 2. The main uncertainty contributors for the NIST SRM are tabulated values for *R*_x_ and *x*_x,b_, because the quantities are given as intervals, and uncertainties were determined as rectangular functions of these intervals. Measuring the isotope ratio of the sample *R*_x_ improved the uncertainty of the NIST SRM to 1.8% instead of 2.6% when using tabulated data for *R*_x_. For avidin, the highest uncertainty contributors were the molar mass of the protein *M* and the mass of dissolved protein *m*. The molar mass was taken from the Uniprot database with an estimated uncertainty of 5%. The mass of avidin had a high relative uncertainty due to the low amount of sample weighed in and the fixed uncertainty of the scale. Moreover, the amount of non-protein-bound sulfur in the avidin solution was much higher than in the BSA solution, resulting in a higher contribution to the uncertainty. However, the uncertainty of the amount of non-protein-bound sulfur also mainly stems from tabulated quantities (see ESI, Fig. S2). The last major uncertainty contributor is the uncertainty of the sulfur mass fraction *w*_x_(S), which mainly consists of the uncertainties of tabulated values for *R*_x_ and *x*_x,b_, and which were also the main contributors to the uncertainty of the NIST BSA protein mass fraction. Hence, besides tabulated quantities, mainly the uncertainty of the mass of avidin weighed into the solution and, to a lower extent, the amounts of sulfur in the digestion and filtration blanks influence the uncertainty of the avidin mass fraction.

**Fig. 3:**
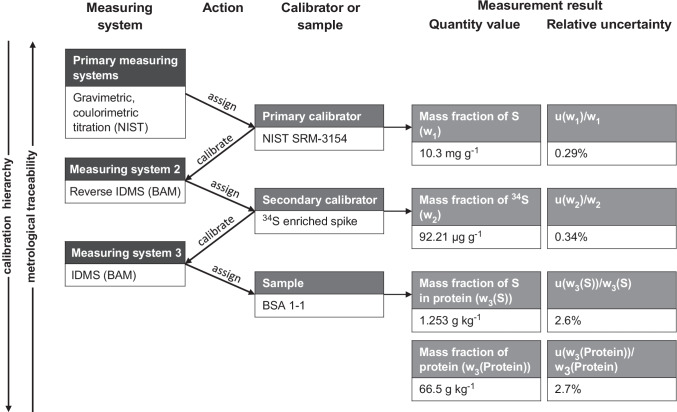
Metrological traceability chain and calibration hierarchy according to IUPAC guidelines [46], shown on the example of BSA sample 1–1

Metrological traceability for the determined protein mass fractions was achieved by using an unbroken chain of calibrations. Figure 3 shows the calibration hierarchy for a representative NIST SRM 927e sample. NIST primary reference measurements serve as the link between the SI and the sulfur mass fraction in the primary calibrator SRM 3154. Reverse ID-ICP-MS using the primary calibrator was then applied to assign the mass fraction of ^34^S to the spike [22, 23]. The spike functioned as a secondary calibrator for the quantification of the sulfur mass fraction. The protein mass fraction was calculated from the sulfur mass fraction and the molar mass of the protein taken from the Uniprot database. Hence, the determined protein mass fraction is traceable to the SI, establishing the complete traceability chain from the kilogram to the sample.

### Comparison with other protein quantification methods

The method developed here was compared with published protein quantification methods on the example of the NIST SRM 927e BSA (Table 5). For comparison, the BSA mass fraction determined by ID-ICP-MS (Table 4) was converted into mass concentration via the given density. Most of the published protein quantification methods are based on AAA, which is an established method and the gold standard in protein quantification. It requires comparatively low amounts of sample and was also used for the traceable quantification of the SRM 927e by NIST. However, AAA requires isotopically labeled amino acid standards as demonstrated by Phinney et al. [24], Wise and Watters [44], and Kinumi et al. [9], whereas ICP-MS–based methods only require an isotopically enriched inorganic spike. AAA also requires hydrolyzing the protein into its amino acids, often followed by the derivatization of amino acids prior to analysis. The optimization of hydrolysis and derivatization conditions is not trivial and may lead to bias. ICP as a (mostly) matrix independent ionization technique requires less sample preparation than AAA, and digestion improves the uncertainty but is not mandatory. Therefore, ICP-MS is a feasible alternative for traceable protein quantification. Nonetheless, AAA is advantageous in terms of pure measurement time and sample consumption. The uncertainties of the BSA concentration determined in this work are slightly higher than uncertainties reported by NIST but in the range of other published methods (see Table 5).

**Table 5:** Comparison of protein quantification methods applied to certified reference materials BSA 927e and 927d

Method	BSA concentration /g/L	Fraction of certified value	Relative uncertainty	Comments	Reference
**Reference material NIST BSA 927e quantified**					
ID-ICP-MS (SF-MS)	67.3 ± 2.0	99.9%	3.6% (*U*, *k* = 2)	**Traceable**	This work
AAA (ID-LC–MS)	67.38 ± 1.38	100%	2.0% (*U*, *k* = 2)	**Traceable**, certified value by NIST, stable isotope amino acids as internal standards	Phinney, Bunk (24)
Biuret method	69.58 ± 1.30	103.3%	1.9% (*U*, *k* = 2)	Reference value by NIST, NIST 927d used as standard	Phinney, Bunk (24)
ID-ICP-MS (QQQ-MS)	67.39 ± 0.40	100.0%	0.6% (SD)		Raeve and Bianga (45)
AAAA (LC-UV)	69.11 ± 3.49	102.6%	5.0% (SD)	Derivatization free, only aromatic amino acids	Hesse and Weller (12)
ES-DMA	65.8 ± 1.6	97.7%	2.4% (SD)	Analysis of droplet entrapped oligomer formation	Li, Tan (47)
**Reference material NIST BSA 927d quantified**					
AAA (ID-LC–MS)	65.41 ± 0.82	100%	1.3% (*U*, *k* = 2)	**Traceable**, certified value by NIST, stable isotope amino acids as internal standards	Wise and Watters (44)
Biuret method	70.10 ± 0.74	107.2%	1.1% (*U*, *k* = 2)	Reference value by NIST, NIST 927c used as standard	Wise and Watters (44)
AAA (ID-LC–MS)	65.4 ± 4.8	100.0%	7.3% (*U*, *k* = 2)	**Traceable**, stable isotope amino acids as internal standards, with pre-column derivatization	Kinumi, Ichikawa (9)
AAA (ID-LC–MS)	66.0 ± 2.4	100.9%	3.6% (*U*, *k* = 2)	Derivatization of multiple functional groups	Sakaguchi, Kinumi (48)
AAA (ID-HILIC-MS)	66.19 ± 2.6	101.2%	4.0% (*U*, *k* = 2)	Stable isotope amino acids as internal standards	Kato, Kato (11)

Raeve and Bianga determined the protein concentration by sulfur ID-ICP-MS using triple quadrupole MS with a collision cell [45]. They also used membrane filtration for the separation of non-protein-bound sulfur comparable to the approach taken in this work. However, they did not calculate an uncertainty budget and did not achieve a traceable result. Moreover, they used 2.5 mg of BSA per measurement and tested the separation after the addition of 5% of non-protein-bound sulfur corresponding to 2.4 µg/g S. Thus, they did not test their method under real-world conditions. In this work, diluted protein solution corresponding to 500 µg of BSA and only 1–2% of non-protein-bound sulfur, corresponding to 70 ng/g S, were used. For the other proteins quantified in this work, as little as 30 µg of total protein was used per analysis. Hence, the here shown method is also applicable for lower amounts of protein as typically handled in quantitative proteomics.

Lee et al. developed an elaborate method for protein quantification based on triple quadrupole sulfur ID-ICP-MS [20]. They separated non-protein-bound sulfur by size-exclusion chromatography and accurately quantified down to 0.09% of non-protein-bound sulfur by ICP-MS using standard addition. By using only 360 µg of protein per analysis, they achieved an expanded uncertainty of 4%, which is in the range of uncertainties reported in this work. The approach shown here is less sophisticated and less powerful because the separation of non-protein-bound sulfur species is limited to small molecules, i.e., contaminants smaller than the membrane cut-off. Nonetheless, the method developed herein is cheaper and easier, and can be applied in any ICP-MS laboratory without hyphenation with a chromatography system and the need for expensive columns. The approach developed in this work is easily applicable and well suited to quantify in-house calibration standards with full SI traceability.

### Application on tau protein and comparison with AAA

The developed method was applied on a pure commercial tau protein for use as a protein calibrator within the ReMiND project. Tau is a biomarker for a group of neurodegenerative diseases denoted “tauopathies,” including Alzheimer’s disease and frontotemporal dementia. The calibration standard was needed for the absolute quantification of tau in the brain of transgenic mice overexpressing human tau, which causes the diseased state in the mice.

The sulfur content in the protein solution was quantified using the optimized ID-ICP-MS procedure and yielded SI-traceable results, which are displayed in Table 6. The sulfur content was corrected for the non-protein-bound sulfur, and the protein mass fraction was determined using the known molar mass and sulfur mass fraction of tau. The protein content in the tau solution was (0.328 ± 0.038) g/kg, which was 30% less than the theoretical amount of protein of (0.475 ± 0.039) g/kg determined from the manufacturer’s information. The deviation might be due to excess moisture and salts, which can cause severe errors in gravimetric measurements. This discrepancy highlights the need for accurate protein quantification of calibration standards as relying on the manufacturer’s information would result in a significant overestimation of the protein content. Moreover, 57% of the total sulfur measured in the protein solution were non-protein-bound sulfur contaminations, which shows that the removal of this fraction is crucial for accurate protein quantification.

**Table 6:** Protein mass fraction in tau protein determined by ID-ICP-MS and corrected for non-protein-bound sulfur. The total sulfur mass fraction in the sample (*w*_x_(S)_total_) and the amount of sulfur in the filtrate (*m*(S)_Filtrate_) were determined by ID-ICP-MS. The total amount of sulfur applied to the filter (*m*(S)_total_) was determined from the mass of sample solution added onto the filter and from *w*_x_(S)_total_. The fraction of non-protein-bound S corresponding to *m*(S)_Filtrate_/*m*(S)_total_ was used to correct the total sulfur mass fraction (*w*_x_(S)_total_). The corrected sulfur mass fraction for protein-bound sulfur (*w*_x_(S)_corr._) was used to calculate the protein mass fraction (*w*_x_(Protein)) in the tau solution. The theoretical amount of tau *w*_x_(Protein)_theor._ was determined from the mass of tau and purity stated by the manufacturer and the mass of solvent. Results with expanded uncertainties *U* with *k* = 2

	*w*_x_(S)_total_ ± *U*/ng/g	*m*(S)_total_ ± *U*/ng	*m*(S)_Filtrate_ ± *U*/ng	Fraction of non-protein-bound S ± *U*/%	*w*_x_(S)_corr._ ± *U*/ng/g	*w*_x_(Protein) ± *U/*g/kg	*w*_x_(Protein)_theor._ ± *U*/g/kg
**Replicates**	4350 ± 160	330 ± 13	196 ± 11	59.3 ± 4.1	1890 ± 190	0.338 ± 0.037	
	4180 ± 160	321 ± 12	178 ± 10	55.3 ± 3.8	1820 ± 180	0.325 ± 0.036	
	4120 ± 160	329 ± 13	181 ± 10	55.0 ± 3.8	1790 ± 180	0.320 ± 0.035	
**Mean**	4217 ± 211	327 ± 14	185 ± 15	56.5 ± 4.8	1833 ± 193	**0.328 ± 0.038**	**0.475 ± 0.039**

The combined uncertainty of the tau protein mass fraction in solution was 11.6% (0.038 g/kg) and was mainly determined by the uncertainties of every single measurement (0.035–0.037 g/kg). The highest uncertainty contributor was the amount of non-protein-bound sulfur. Analogous to avidin, this uncertainty mainly stemmed from tabulated quantities. Only 9% of the uncertainty, stemming from the mass of the blank filtrate, consisted of measured data. The highest uncertainty contributions stemmed from tabulated values for the isotope ratios *R*, the isotope amount fractions *x,* and the molar mass *M* (see ESI, Fig. S3). This shows that the procedure is sufficiently optimized and that the high uncertainties of tabulated intervals for isotope ratios, isotopic abundances, and molar mass are the limiting factors for the total uncertainty. Measuring these values would decrease the final uncertainty of the determined protein mass fraction. However, a higher amount of sample is needed for reliable determination of these quantities, which is often not available for proteins.

For additional validation besides the previously described BSA analysis, the protein concentration in the tau solution was also quantified by AAAA via its tyrosine content and was determined as (0.309 ± 0.040) g/L. The calibration data and tyrosine concentration are shown in the ESI (Sect. 1.2). For comparison with the ID-ICP-MS result of (0.328 ± 0.038) g/kg, the quantified amount of tau was converted from mass concentration to mass fraction (see ESI, Sect. 1.2), resulting in a mass fraction of (0.309 ± 0.040) g/kg. To assess the metrological compatibility of the AAAA and the ID-ICP-MS result, the *E*_n_ value was calculated and was determined to be 0.17. This indicates that the difference between the values is less than 20% of the corresponding uncertainty and that the values agree well. Hence, commercially available proteins can be reliably quantified at sufficiently low concentrations for real-world applications, e.g., as calibrators.

## Conclusion

For conventional protein quantification methods, reliable protein standards are required, but commercially available proteins are usually not sufficiently characterized. Thus, accurate quantification of commercial proteins for use as calibrators is crucial for all subsequent analyses because manufacturers often overestimate the protein content in their products, resulting in deviations up to 30%. Moreover, quantification needs to be done in solution because residual buffers, stabilizers, and other additives can comprise a considerable amount of the dry weight. Hence, we developed and optimized a method for quantitative proteomics, which can be used to characterize SI-traceable in-house standards in solution. The method is based on ID-ICP-MS for quantification of the sulfur content of a protein solution and the amount of non-protein-bound sulfur separated by membrane filtration. After correcting for non-protein-bound sulfur contaminations, we determined the protein mass fraction of the protein solution from the known sulfur mass fraction. Separation of non-protein-bound sulfur was found to be essential for accurate quantification as contaminations can easily comprise more than 50% of the total sulfur content. We successfully applied the method for quantification of a tau protein solution for use as a calibration standard. The method proved to be easily applicable, fast, and comparatively cheap, and uses reasonable amounts of protein material while retaining SI traceability with good uncertainties comparable to other established methods.

The BSA protein content is given here in mass concentration (g/L) and in Table 4 in mass fraction (g/kg), causing differing numerical values.

*U* expanded uncertainty; *SD* uncertainty given is only the standard deviation of repeated measurements, and no uncertainty budget was determined; *AAAA* aromatic amino acid analysis (by molecular mass spectrometry); *ID* isotope dilution; *LC* liquid chromatography; *MS* mass spectrometry; *ICP* inductively coupled plasma (ionization for elementary mass spectrometry); *SF* sector field; *QQQ* triple quadrupole; *ES-DMA* electrospray differential mobility analysis; *HILIC* hydrophilic interaction liquid chromatography.

## Supplementary Information

Below is the link to the electronic supplementary material.

Supplementary file1 (DOCX 52.2 KB)

## Data Availability

Not applicable.
