# Supplementary material for: Procedure providing SI-traceable results for the calibration of protein standards by sulfur determination and its application on tau
Source: Anal Bioanal Chem. 2022 Mar 22;414(15):4441–55. doi: 10.1007/s00216-022-03974-z (PMC9142460; doi:10.1007/s00216-022-03974-z)
Supplement: Supplementary file 1 — Supplementary file1 (DOCX 52.2 KB) [file 216_2022_3974_MOESM1_ESM.docx]

Electronic Supplementary Information to

Procedure providing SI-traceable results for the calibration of protein standards by sulfur determination and its application on tau

Nora Lemke^1,2^, Ahmed H. El-Khatib^1,3^, Teodor Tchipilov^1^, Norbert Jakubowski^4^, Michael G. Weller^1^, Jochen Vogl^1§^

^1^ Bundesanstalt für Materialforschung und -prüfung (BAM), Richard-Willstätter-Straße 11, 12489 Berlin, Germany

^2^ Charité - Universitätsmedizin Berlin, Hessische Str. 3-4, 10115 Berlin, Germany

^3^ Department of pharmaceutical analytical chemistry, Faculty of Pharmacy, Ain Shams University, Cairo, Egypt

^4^ Spetec GmbH, Am Kletthamer Feld 15, 85435 Erding, Germany

^§^ corresponding author

# Materials and methods

## Calculation of the *E_n_* value

The *E*_n_ value is calculated with

$E_{n}= \frac{\left| d_{ij} \right|}{U(d_{ij})}$ = $\frac{\left| x_{i}-x_{j} \right|}{k\cdot u\left( d_{ij} \right)}$

and

$$u\left( d_{ij} \right)= \sqrt{u^{2}\left( x_{i} \right)+u^{2}\left( x_{i} \right)-2\cdot\mathrm{cov}(x_{i},x_{j})}$$

with *d_ij_* being the difference between two metrological values *x_i_* and *x_j_* and *U(d_ij_)* the expanded uncertainty of the difference. *U(d_ij_)* is calculated using the combined uncertainty *u(d_ij_)* from the uncertainties *u(x_i_)* and *u(x_j_)* of the compared values and their covariance cov(*x_i_*,*x_j_*). The values *x_i_* and *x_j_* are metrologically compatible when the corresponding *E*_n_ value is ≤ 1.

## Amino acid analysis of tau protein

For validation, the tau protein concentration was determined by AAA via the content of the aromatic amino acid tyrosine (Tyr). Tyr was quantified via the area of its UV emission peak after hydrolysis of the tau protein samples. Integrated peak areas were averaged, and the Tyr concentration was determined by external calibration using a commercial amino acid standard. The linear calibration curve had a slope of (32.58 ± 0.13) L/nmol and a y-intercept of 19.0 ± 24.0, yielding mean Tyr concentrations of (32.4 ± 3.9) nmol/L in stock. Recoveries of (96 ± 1) % were previously determined for tyrosine, resulting in corrected tyrosine concentrations of (33.7 ± 4.3) nmol/L in stock, as shown in Table S4. The tau concentration in solution was determined as (0.309 ± 0.040) g/L.

For comparison with tau protein mass fraction by ID-ICP-MS, the AAA result had to be converted from g/L to g/kg via the density of the sample solution. The density of mixed materials cannot be easily calculated and was, therefore, estimated to (1000 ± 5) g/L from the densities of the salt, the water, and the protein in the solution. Tau protein with the determined concentration of ~0.3 g/kg was diluted in 50 mmol/L NH_4_HCO_3_. Pure water has a density of 997.77 g/L at 22 °C; the protein contributes roughly 0.3 g/L and 0.05 mol/L of NH_4_HCO_3_ with a molar mass of 79 g/mol result in an additional density contribution of 3.95 g/L. By adding these factors, a density of 1002 g/L is obtained. However, mixing the solution is not a completely linear process because effects such as volume contraction might influence the final volume and density of the mixture. Therefore, a density of (1000 ± 5) g/L was estimated for the protein solution and used for the calculation of the tau protein mass fraction from the concentration determined by AAA.

# Tables

Table S1: Instrumentation and operating conditions used for sulphur isotope dilution measurements.

| **Parameter** | **Setting** |
| --- | --- |
| Instrument type | Element 2 |
| Autosampler | Cetac ASX-100 |
| Nebulizer | MicroMist 100 µL |
| Spray chamber | Cyclonic |
| Interface | Jet interface |
| Cones | Ni sampler and skimmer cones |
| Cool gas flow rate | 16 L/min Ar |
| Auxiliary gas flow rate | 0.9 – 1.2 L/min Ar |
| Sample gas flow rate | 1.0 – 1.25 L/min Ar |
| RF power | 1250 W |
| Mass resolution mode | Medium (R = 4000) |
| Acquisition mode | pulse and analog |
| Runs/passes | 20/100 |
| Mass window | 125% |
| Integration window | 60% |
| Samples per peak | 30 |
| Sample time | 10 ms |

Table S2: Quantities for ID-ICP-MS calculation with given uncertainties.

| **Symbol** | **Sample** | **Value** | **Uncertainty** | **Distribution** | **Source** |
| --- | --- | --- | --- | --- | --- |
| *w*_y,b_ | all | (200 - 20,000) ng/g | 0.2 % | normal (*k* = 2) | Ref. [1] |
| *x*_x,b_ | protein | 0.042192 mol/mol | 0.000621 | rectangular | CIAAW |
|  | unknown | 0.04189 mol/mol | 0.001282 | rectangular | CIAAW |
| *M*_x_ | protein | 32.064368 | 0.001308 | rectangular | CIAAW |
|  | unknown | 32.063732 | 0.002703 | rectangular | CIAAW |
| *M*_b_ | all | 33.96786687 | 0.00000028 | normal (*k* = 2) | Ref. [1] |
| *m*_y_ | all | (0.08 - 5) g | 0.0001 g | normal (*k* = 1) | measured |
| *m*_x_ | all | (0.08 - 5) g | 0.0001 g | normal (*k* = 1) | measured |
| *R*_y_ | all | 0.002120 | 0.000056 | normal (*k* = 2) | Ref. [1] |
| *R*_x_ | protein | 22.5251 | 0.3472 | rectangular | CIAAW |
|  | unknown | 22.7123 | 0.7283 | rectangular | CIAAW |
| *R*_xy_ | all | ~ 1 | (0.1 - 0.3) % | normal (*k* = 1) | measured |

Table S3: Relative uncertainties of protein mass fractions. *U*: expanded uncertainty *k* = 2, σ: standard deviation, *u*_c_: combined uncertainty. BSA: NIST SRM 927e.

|  | **Relative uncertainty of *w*_x_(Protein) / %** | | | |
| --- | --- | --- | --- | --- |
|  | *U* of a single measurement | *σ* of all measurements | *u*_c_ of all measurements | *U* of all measurements |
| **BSA** | 2.6 | 2.1 | 1.5 | 3.1 |
| **Avidin** | 8.3 | 3.5 | 4.6 | 9.2 |

Table S4: Quantified tau protein concentration by AAAA. *u*: standard deviation of the mean. *U*: expanded uncertainty at 95 % confidence with *k* = 2 calculated by GUM. The concentration of tyrosine (Tyr) in dilution was calculated after blank correction.

|  | **Area ± *u* / a.u.** | | ***c*(Tyr) ± *U* / nM** | ***c*(Tyr) ± *U* / µM** | ***c*(Tau) ± *U /* g/L** |
| --- | --- | --- | --- | --- | --- |
|  | replicates | mean | in dilution | in stock |  |
| **Tau** | 30210 | 32415 ± 1803 | 930 ± 110 | 33.7 ± 4.3 | 0.309 ± 0.040 |
|  | 31047 |  |  |  |  |
|  | 35988 |  |  |  |  |
| **Blank** | 2246 | 2241 ± 6 | - | - | - |
|  | 2235 |  |  |  |  |

# Figures

## Uncertainty contributors

Figure S1: Contributors to the uncertainty of the BSA mass fraction. *R*_x_(Protein, CIAAW) and *x*_x,b_(Protein, CIAAW) were taken from tabulated data published by CIAAW. The quantities are given as intervals, and uncertainties were determined as rectangular functions of these intervals.

Figure S2: Final uncertainty contributions of the avidin protein mass fraction after including uncertainty contributors for the fraction of non-protein bound sulfur *f* and the mass fraction of sulfur in the protein solution *w*_x_(S). Isotope ratios *R*, isotope amount fractions *x,* and molar mass *M* were taken from tabulated data.

Figure S3: Final uncertainty contributions of the tau protein mass fraction.

# References

1. Pritzkow W, Vogl J, Köppen R, Ostermann M. Determination of sulfur isotope abundance ratios for SI-traceable low sulfur concentration measurements in fossil fuels by ID-TIMS. International Journal of Mass Spectrometry. 2005;242(2):309-18. <https://doi.org/10.1016/j.ijms.2004.10.024>.
